# Assessing drivers of localized invasive spread to inform large‐scale management of a highly damaging insect pest

**DOI:** 10.1002/eap.2538

**Published:** 2022-02-20

**Authors:** Gabriela C. Nunez‐Mir, Jonathan A. Walter, Kristine L. Grayson, Derek M. Johnson

**Affiliations:** ^1^ Department of Biology Virginia Commonwealth University Richmond Virginia USA; ^2^ Department of Biological Sciences University of Illinois at Chicago Chicago Illinois USA; ^3^ Department of Environmental Sciences University of Virginia Charlottesville Virginia USA; ^4^ Department of Biology University of Richmond Richmond Virginia USA

**Keywords:** biological invasions, climate suitability, invasive management, invasive species, invasive spread, large‐scale management, *Lymantria dispar*, pest management

## Abstract

Studies of biological invasions at the macroscale or across multiple scales can provide important insights for management, particularly when localized information about invasion dynamics or environmental contexts is unavailable. In this study, we performed a macroscale analysis of the roles of invasion drivers on the local scale dynamics of a high‐profile pest, *Lymantria dispar dispar* L., with the purpose of improving the prioritization of vulnerable areas for treatment. Specifically, we assessed the relative effects of various anthropogenic and environmental variables on the establishment rate of 8010 quadrats at a localized scale (5 × 5 km) across the entire *L. dispar* transition zone (the area encompassing the leading population edge, currently from Minnesota to North Carolina). We calculated the number of years from first detection of *L. dispar* in a quadrat to the year when probability of establishment of *L. dispar* was greater than 99% (i.e., waiting time to establishment after first detection). To assess the effects of environmental and anthropogenic variables on each quadrat's waiting time to establishment, we performed linear mixed‐effects regression models for the full transition zone and three subregions within the zone. Seasonal temperatures were found to be the primary drivers of local establishment rates. Winter temperatures had the strongest effects, especially in the northern parts of the transition zone. Furthermore, the effects of some factors on waiting times to establishment varied across subregions. Our findings contribute to identifying especially vulnerable areas to further *L. dispar* spread and informing region‐specific criteria by invasion managers for the prioritization of areas for treatment.

## INTRODUCTION

Biological invasions are one of the major ecological threats in the Anthropocene, making this phenomenon a major priority in ecological research and ecosystem management (Pyšek & Richardson, 2010; Simberloff et al., 2013; Vitousek et al., 1997). Invasion research benefits from a macrosystems ecology perspective as comprehensive, large‐scale databases become more available and analytical methods become more advanced (Fei et al., 2016). Through this framework, ecologists can address ecological questions and environmental problems through a multi‐scale lens by studying patterns and processes at macroscales (i.e., regional to continental), while incorporating effects and interactions of processes occurring at more localized scales. Macroscale studies and studies adopting a multiscale framework are especially important in invasion research, as drivers of invasion occur at various scales over vast spatial extents (e.g., trade, human travel, land‐use; Liu et al., 2013). Invasion management may also benefit from the findings produced by multi‐scale studies, as landscape‐level knowledge is often utilized when localized information about the dynamics of specific invasive species or specific environmental contexts are unavailable (Blackwood et al., 2010; Coutts et al., 2011; Fletcher & Westcott, 2013).

In this study, we seek to provide new insights on macroscale drivers of invasive spread and their implications for management, focusing on *Lymantria dispar dispar* L. (previously known as European gypsy moth). *Lymantria dispar* is a polyphagous foliage‐feeding insect that commonly reaches outbreak densities, resulting in widespread tree defoliation and cascading ecological effects, including altered species compositions and nutrient cycles (Gandhi & Herms, 2010; Lovett et al., 2006). Introduced in Massachusetts, USA in 1869, *L. dispar* is now established in 20 US states and five Canadian provinces (Animal and Plant Health Inspection Service, 2019) and has caused the defoliation of >98 million acres of forest since 1924 (USDA Forest Service, 2020a). This species is a high‐profile invasive insect pest and model system for invasion ecology and science‐based invasion management based on its damaging periodic outbreaks, extensive introduced range in Eastern North America, and long‐term fine‐scale trapping effort that covers the entirety of the US transition zone at the front and ahead of the leading edge of the invasion.

Management of *L. dispar* spread and impacts is coordinated by the USDA Gypsy Moth Management program, which comprises various projects, including the National Gypsy Moth “Slow the Spread” program (STS; USDA Forest Service, 2020b). Currently, the STS program focuses on suppressing or eradicating nascent local populations along the expanding population front that are identified using an extensive trapping network and a decision‐making algorithm based on trap‐catch count data. This trapping network includes >80,000 traps deployed every year (Tobin, 2008). However, it is often the case that the number of “potential problem areas” identified in a given year is greater than what can feasibly be managed, making prioritization of problem areas a necessity (Tobin & Sharov, 2007). An understanding of the environmental and anthropogenic drivers of localized spread of *L. dispar* across the range could enhance prioritization of treatment areas when combined with the STS program decision algorithm.

Given the detailed spatiotemporal data available for *L. dispar*, studies on this species have substantially advanced our understanding of invasion dynamics and pest management. While a number of studies have examined large‐scale patterns of spread in this species (e.g., Bjørnstad et al., 2010; Johnson et al., 2006; Liebhold et al., 1992; Régnière & Sharov, 1999; Tobin et al., 2014), important gaps in our understanding remain. Previous research has examined the roles of various environmental and anthropogenic factors (e.g., temperature, precipitation, host tree abundance) in *L. dispar* biology and invasion dynamics (Alalouni et al., 2013; Bigsby et al., 2011; Doane & McManus, 1981). However, these factors have mostly been studied separately (exceptions include Sharov et al. [1999] and Lippitt et al. [2008]) and the majority of studies use fine spatial and temporal scales (with some exceptions referenced above). As a result, we are currently unable to discern the relative roles of environmental and anthropogenic factors at local scales, and how these roles change across the invasive range. This information is necessary to obtain a better understanding of drivers of *L. dispar* invasion and associated spatial patterns, which could allow us to identify the habitat characteristics in a given geographical context that make certain areas especially vulnerable to its invasion.

Here, we address this gap by performing a macroscale investigation of the effects of a wide range of anthropogenic and environmental factors on the rate at which *L. dispar* became established in 5 × 5 km quadrats (i.e., the waiting time from first detection to establishment) across the *L. dispar* transition zone—the region between infested and uninfested areas along the population leading edge. Additionally, to explore whether these drivers change across geographical areas, we assessed the effects of these factors for three separate subregions with distinct environmental and management contexts. This study was performed using a multi‐decadal database (1985–2015) of *L. dispar* trap‐catch data compiled by the STS program. The research presented here demonstrates the utility of macroscale research to large‐scale science‐based invasive management by presenting an expedient approach to assessing the environmental and anthropogenic factors driving localized spread of invasive species across broad geographical regions. Our findings provide timely insights into the processes influencing *L. dispar* invasion across the US invasive range that can be applied to the prioritization of *L. dispar* treatments, as well as to predict future hotspots with high potential for establishment and population growth. These insights could also be applicable and greatly useful for large‐scale management of other invasive insects.

## METHODS

### Data set

The negative ecological and economic impacts of *L. dispar* outbreaks have resulted in a large‐scale effort to collect comprehensive data on spread rates for research and management purposes (Grayson & Johnson, 2018; Tobin et al., 2004). The STS program monitors abundances along the transition zone using a network of tens of thousands of traps deployed annually (over 80,000 traps) (Tobin, 2008; Tobin & Blackburn, 2007). The transition zone presently spans 11 states, from North Carolina to Minnesota. The transition zone has been monitored since 1985 by various programs (by the AIPM program from 1985 to 1992, the STS pilot program from 1992 to 2000, and presently by the STS program since 2000) via the deployment of pheromone‐baited traps generally distributed on grids with traps spaced 0.5–2 km apart, with traps more sparsely distributed in the infested areas behind the leading edge (trap spacing 3–8 km) (Tobin & Blackburn, 2007). We spatially gridded the transition zone into 5 × 5 km (25 km^2^) quadrats in ArcGIS, and then obtained the median number of *L. dispar* moths caught in all traps within each quadrat from 1985 to 2015. Prior to gridding the data, we omitted traps within 1.5 km of an area treated for *L. dispar*. Removing traps within a 1.5 km radius of treated areas is standard practice in studies using STS trap catch data (e.g., Tobin et al., 2007), as this distance (1.5 km) is longer than the expected drift of *L. dispar* treatments (Teschke et al., 2001), as well as beyond the distance of natural dispersal of *L. dispar* moths (Robinet et al., 2008; Walter et al., 2016). In practice, <2% of the monitoring area was treated in any given year.

### Calculating waiting times to establishment

In this study, we use the establishment rate of 5 × 5 km quadrats as a measure of spread at the landscape scale (i.e., combination of local dispersal and population growth). Landscape spread of invasive species depends on the processes of local dispersal, habitat connectivity, and establishment (Theoharides & Dukes, 2007). After a species arrives at a novel location through dispersal, it may or may not become established. Establishment occurs if the population grows to a size where it is unlikely to go locally extinct. This process of localized range expansion is conceptually similar to the dispersal and population growth components of Skellam's model of diffusive spread of biological invasions (Skellam, 1951). We measured establishment rate as the time elapsed between initial detection and establishment of *L. dispar* in a quadrat, in other words, the waiting time to establishment. The concept of “waiting time” has emerged relatively recently in invasion research as a time‐to‐event approach to measuring or predicting the rate at which various invasion processes occur, such as introduction (Jerde & Lewis, 2007), establishment (Drake & Jerde, 2009; Mang et al., 2017), competitive domination (Korniss & Caraco, 2005; Yamaguchi et al., 2019), and spread (Goldstein et al., 2019).

To calculate waiting time to establishment, it was first necessary to define the conditions by which *L. dispar* would be considered established in a quadrat. Generally, previous research on *L. dispar* invasion has determined establishment status based on a population density threshold (Walter, Johnson, et al., 2015a; Walter et al., 2016). However, that approach ignores inherent uncertainties due to measurement error, demographic stochasticity, latent dispersal, and Allee effects. Each of these factors can cause population densities to cross over or under a threshold population density multiple times, leading to noisy and uncertain invasion estimates. Here, we seek to mitigate these limitations by using a probabilistic framework to determine establishment status, an innovative approach in this context. In this framework, the probability that a quadrat would have no moths in a given year was used as the criterion to consider a quadrat as not established by *L. dispar*. Correspondingly, one minus the probability of no moths was used to determine *L. dispar* establishment.

**FIGURE 1:**
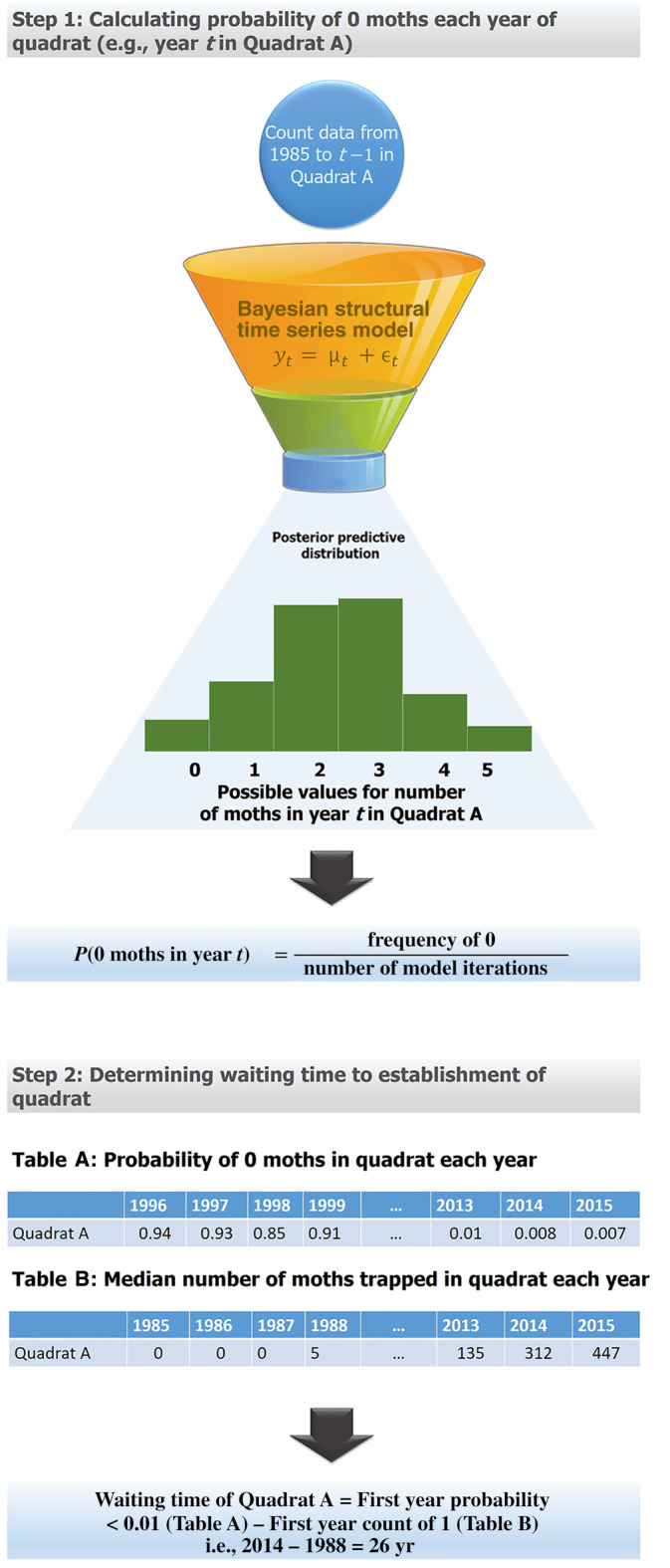
Flowchart detailing the methodological process followed to obtain the waiting time to establishment of each quadrat in the *Lymantria dispar* transition zone. In the Bayesian structural time series model equation, *y*_𝑡_ represents the estimated value for year *t*, *μ*_𝑡_ represents the trend component derived from the observed data, and ϵ_𝑡_ represents a Gaussian i.i.d. error term

We followed a multi‐step methodology featuring Bayesian structural time series models (R package *bsts*; Scott, 2017; Figure 1). First, we used these time series models to calculate the probability that traps in each quadrat captured a median of zero moths each year. We selected the median instead of other measures (i.e., sum or mean) because it is insensitive to both variation in number of observations and non‐normal distributions. As in traditional time series models, our Bayesian structural time series models were calibrated to forecast the number of *L. dispar* moths in a quadrat in year *t* as a function of the annual moth counts in the preceding years (i.e., from *t* − *n* to *t* − 1).

We then used the Bayesian posterior distribution of latent moth density to calculate the probability (*p*) that a quadrat was uninvaded that year. Specifically, we calculated the probability that there was a median of zero months in a quadrat in a given year, that is, that the latent population density in said quadrat is below that which any moths would be trapped. Probability of establishment for each quadrat in a given year was therefore defined as 1 − *p*. Waiting time for a quadrat was defined as the number of years between the year of initial detection (i.e., first year in which a median of one *L. dispar* moth was recorded for said quadrat) and the year of establishment (i.e., first year in which probability of zero moths was <0.01). Following the general guidance on statistical confidence intervals, we chose this threshold as a conservative definition for probability of establishment. The predictive value of the time series model (i.e., model fitness to the data) was assessed by correlating the forecasted number of moths in a quadrat to the observed number each year (these model validation results can be found in Appendix S1).

**FIGURE 2:**
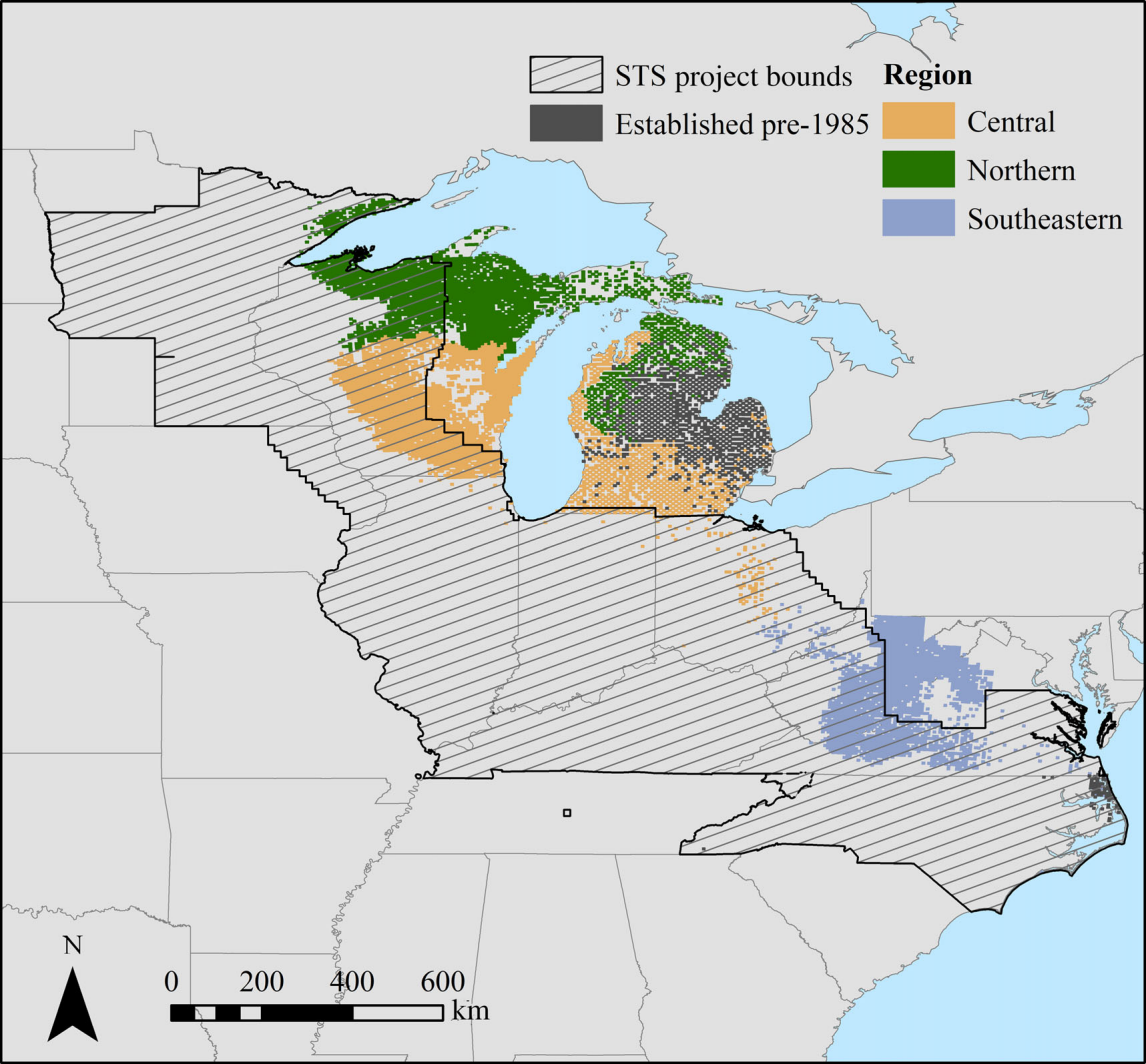
Subregions within the *L. dispar* transition zone based on level II eco‐regions and quadrats with data used in this study (*n* = 8010). Each color indicates the subregion within which each quadrat is found: northern mixed wood shield subregion (*n* = 2642), central plains subregion (*n* = 3120), and southeastern forests and plains subregion (*n* = 2245)

Since we had annual *L. dispar* moth counts from 1985 to 2015, we used the first 10 years to calibrate our models. Doing so ensured we had at least 10 years of time series data for each of the time series models used to calculate establishment probability (1995–2015). We excluded quadrats that were clearly not established by *L. dispar* by 2015 (i.e., quadrats with less than 10 moths caught between 1985 and 2015) and quadrats that were likely to have been already established when traps were first deployed (i.e., quadrats whose first *L. dispar* count on record was more than zero). Out of the 55,395 quadrats throughout the *L. dispar* transition zone, 8010 quadrats changed from uninvaded to established between 1985 and 2015, and therefore were selected for further analysis. To explore spatial patterns across the range, we defined three subregions based on level II eco‐regions: northern mixed wood shield, central plains, and southeastern forests and plains (Omernik, 1995; Omernik & Griffith, 2014; Figure 2). These subregions comprise areas with similar environmental contexts (e.g., climate, biota) and are relevant to management, given the organization of management units distributed across the range.

### Assessing influence of environmental and anthropogenic factors

In order to identify environmental and socioeconomic factors that may affect establishment rates, we compiled data on both known and potential drivers of *L. dispar* population growth and local spread (Table 1). We focused on factors associated with population growth and factors associated with short‐range anthropogenic movement of propagules and natural local dispersal. Most of these factors have been shown to have an effect on the biology and ecology of *L. dispar*, while others are known general drivers of invasions (see references in Table 1). Using ArcMap (ArcGIS, version 10.7; ESRI, Redlands, California, USA), we obtained the mean value of each factor within each 5 × 5 km quadrat. Our climate variables consisted of a 20‐years average for each quadrat across the years 1995 to 2015.

**TABLE 1:** Source and resolution of variables included in the range‐wide and subregional models

Category and Variable	Description	Source	Resolution	Reference
Population growth				
Winter temperature (°C)	Across years average of mean temperature in January	PRISM Climate Group	30 arc sec	Liebhold et al. (1992)
Maximum spring temperature (°C)	Across years average of maximum temperature in May	PRISM Climate Group	30 arc sec	Leonard (1981)
Summer precipitation (mm)	Across years average of mean precipitation in July	PRISM Climate Group	30 arc sec	Leonard (1981)
Elevation (m)		USGS Digital Elevation Models	1/3 arc sec	Sharov et al. (1997)
Host abundance	Average basal area of host trees	Morin et al. (2005)	1 km^2^	Sharov et al. (1997)
Anthropogenic movement				
Population density 2010 (individuals/km^2^)		Iannone III et al. (2015)	County	Lippitt et al. (2008)
Wood use	Number of housing units that use wood for heating purposes	2016 TIGER/Line Census	Block groups	Bigsby et al. (2011)
Highway density (km/km^2^)		2000 TIGER/Line Census	County	Lippitt et al. (2008)
Natural dispersal				
Average anthropogenic fragmentation	Average proportion of adjacent pixel pairs in which one pixel is forest and the other anthropogenic land use	Riitters et al. (2000)	9 km^2^	(Iannone III et al. (2015)
Average natural fragmentation	Average proportion of adjacent pixel pairs in which one pixel is forest and the other a natural land cover type	Riitters et al. (2000)	9 km^2^	Iannone III et al. (2015)

We performed mixed‐effects linear regression models for all geographical subregions, including a transition zone‐wide model (hereafter referred to as “range‐wide” for expediency). In addition to the factors in Table 1, we also included two “background” variables that are known to have a strong influence on *L. dispar* local establishment and spread, and are already accounted for in the STS decision algorithm, directly or indirectly (Tobin & Sharov, 2007). These variables are: (1) the year of first detection of *L. dispar* in a quadrat and (2) the average waiting time to establishment for the surrounding 3 × 3 quadrat neighborhood. We included year of first detection (i.e., the first year in which a median of at least one *L. dispar* moth was recorded for a quadrat) in the model to account for spatiotemporal patterns in *L. dispar* spread. Averaged waiting time for the surrounding area was added for two reasons. First, we wanted to focus on the ability of local characteristics to produce local hot/cold spots of invasion (i.e., accelerations or retractions of establishment rate) that depart from the neighborhood average. Second, we wanted to account for propagule pressure from other invaded quadrats, a major predictor of establishment probability currently used in the prioritization algorithm. Finally, we included level IV eco‐regions (a more fine‐scaled classification than the level II eco‐regions used to define subregions) as a random effect to account for spatial autocorrelation and geographic latent factors.

For each subregion and for the full transition zone, we fitted all possible model combinations using the dredge function implemented by R package *MuMIn* (Barton, 2009). We then selected the model with the lowest Akaike's information criterion (AIC) score. Although we consider some candidate predictors that tend to be collinear, because AIC penalizes for increasing model complexity and collinear predictors contain redundant information, models containing collinear predictors will tend to perform poorly compared to informative models with low collinearity among predictor variables. As a check, we examined variance inflation factors (VIF) of the selected models that minimized AIC. All VIF values were well below the established guidance value of 10 (Miles, 2014), indicating that serious multicollinearity is unlikely (Appendix S2: Table S1).

In order to evaluate the performance of these models, we calculated three measures of *R* ^2^. Traditional measures of *R* ^2^ are not well‐suited to measure goodness‐of‐fit of mixed‐effects models, as these models differ from linear models in that they have additional components of variance. For that reason, we calculated two measures appropriate for mixed‐effects models: marginal *R* ^2^ (variance explained by the fixed effects) and conditional *R* ^2^ (variance explained by both fixed and random effects; Nakagawa & Schielzeth, 2013). We used the functions r.squaredGLMM from R package *MuMIn* (Barton, 2009) and r2beta from R package *r2glmm* (Jaeger, 2017) to derive the marginal and conditional *R* ^2^ of each model. We also calculated the marginal *R* ^2^ for all environmental and anthropogenic factors combined (i.e., excluding average waiting time of neighborhood and year of initial detection).

## RESULTS

**FIGURE 3:**
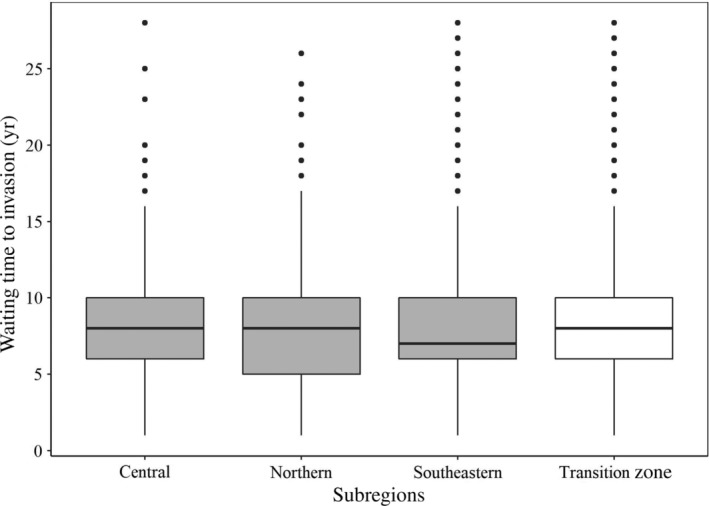
Box plot of waiting times to invasion of 5 × 5 km quadrats invaded by *L. dispar* across the transition zone (i.e., full range) (*n* = 8010), and across three subregions within the zone: Northern mixed wood shield subregion (*n* = 2642), Central plains subregion (*n* = 3120), and Southeastern forests and plains subregion (*n* = 2245). Each box in these box plots represents the interquartile range of the distribution of waiting times from the 25th to 75th percentiles, with the midline representing the median. The whiskers in each box plot extend to the minima and maxima. Dots represent outliers

**FIGURE 4:**
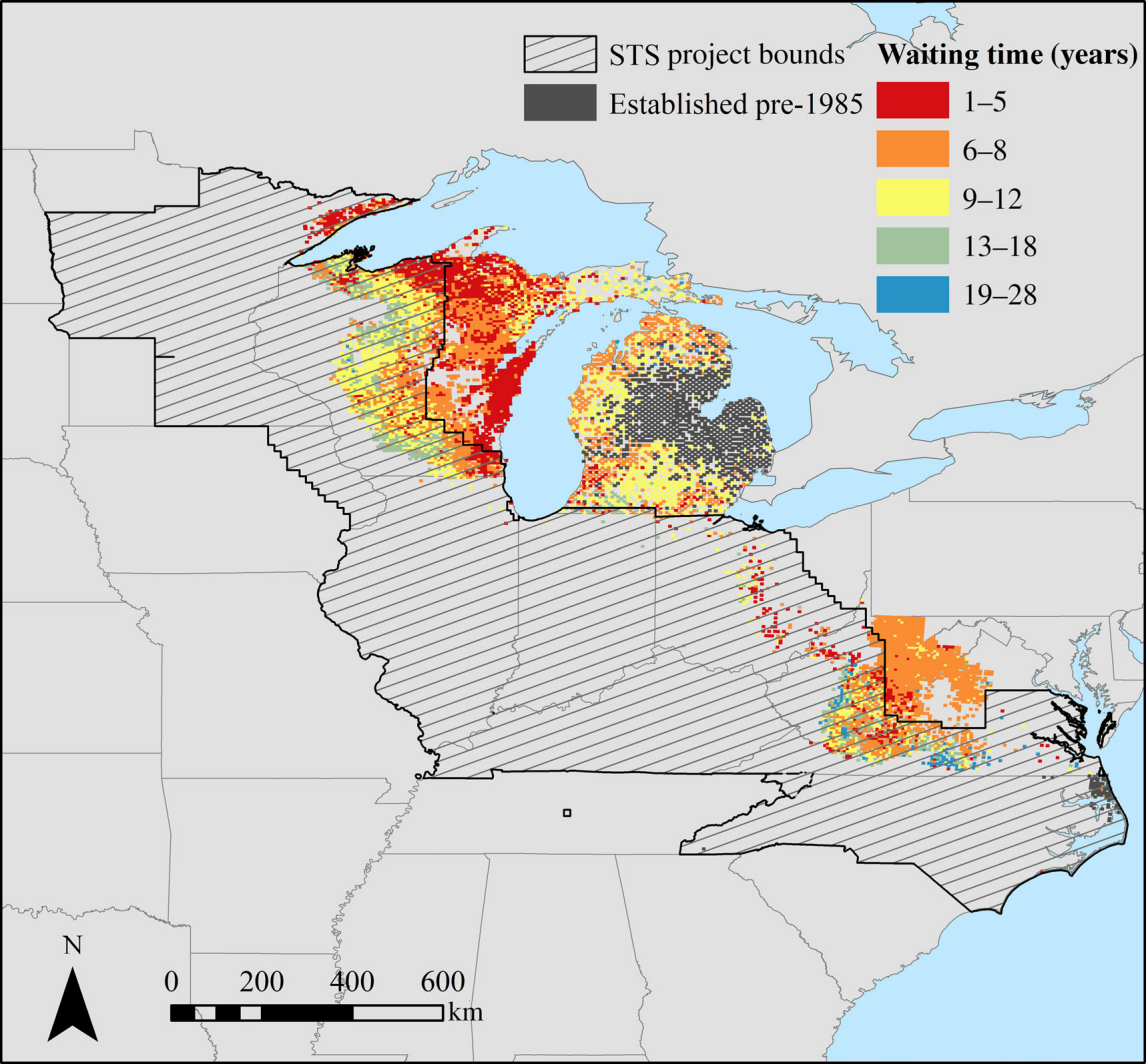
Number of years from first *L. dispar* detection to establishment for all invaded quadrats (*n* = 8010) across the *L. dispar* transition zone. Color of each quadrat indicates the waiting time to establishment calculated for the quadrat. Dark gray coloring indicates quadrats with non‐zero trap catch when monitoring started (1985). These quadrats were considered to be established prior to monitoring and excluded from analyses. STS project bounds (i.e., areas where monitoring and treatment have occurred from 2007 to 2015) are indicated by a solid black line and black diagonal stripes

The average waiting time to establishment for fine‐scale quadrats across the transition zone was 7.9 years, a pattern that was mostly consistent across subregions (northern, 7.5 years; central, 8.1 years; southeastern, 8.2 years; Figure 3). Nonetheless, waiting times to establishment did display other spatial patterns. Waiting times across the transition zone appeared to be progressively longer at the leading edge of the invasion, suggesting that the rate of establishment, and therefore the rate of localized spread, is decreasing over time (Figure 4).

**FIGURE 5:**
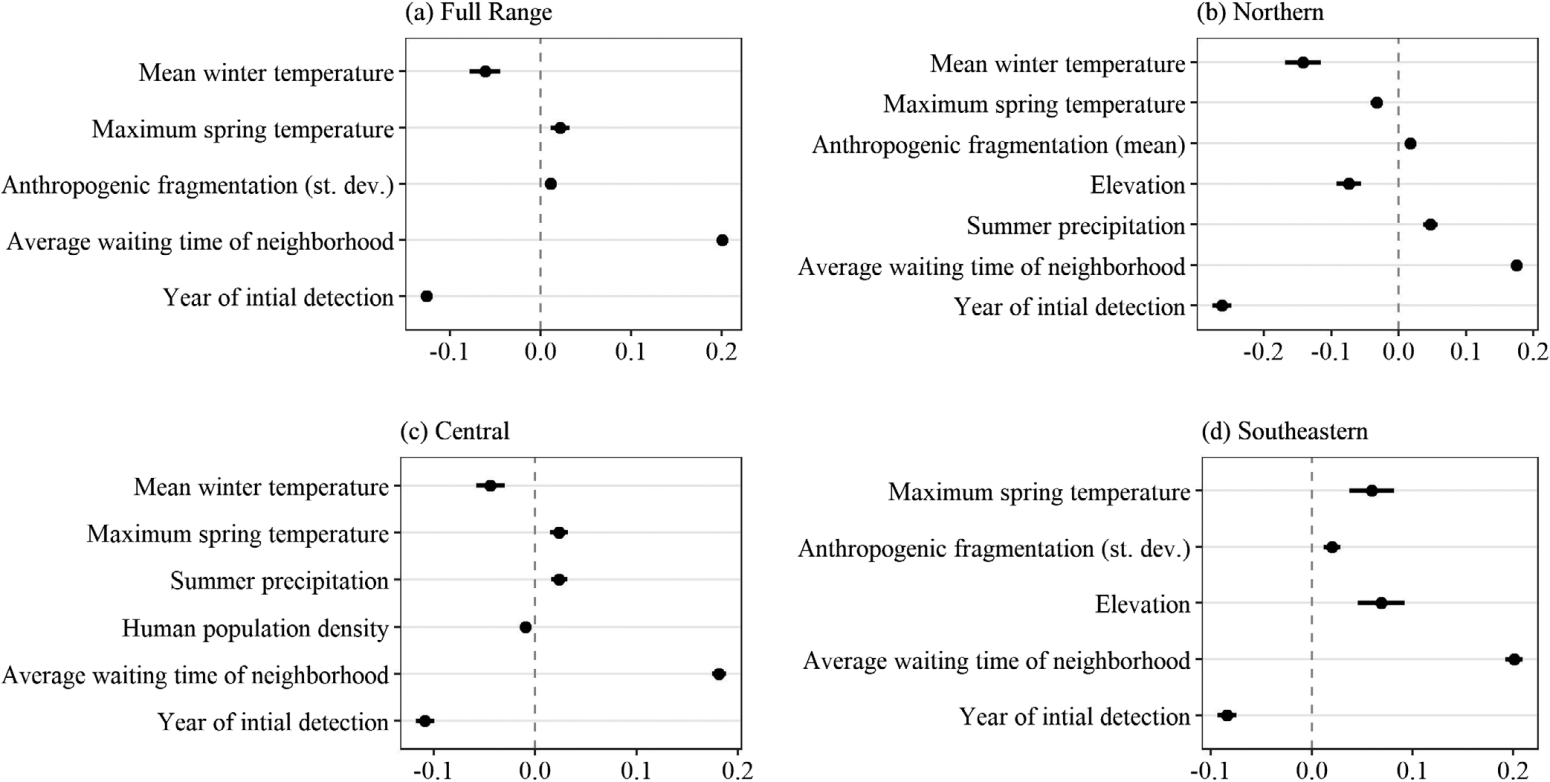
Fixed‐effects results of four linear mixed‐effects regression models: (a) full range‐wide model (*n* = 8010), (b) northern mixed wood shield subregion (*n* = 2642), (c) central plains subregion (*n* = 3120), and (d) southeastern forests and plains subregion (*n* = 2245). Dots and whiskers depict the coefficient estimate and standard error for 10 environmental and anthropogenic drivers, as well as two background variables (i.e., year of initial detection and average waiting time of neighborhood). See Appendix S3 for full detailed results. Avg., average

As expected, the background variables, average waiting time of neighborhood and year of first detection, had the strongest effects on waiting time to establishment across all models, underlining the importance of taking these factors into account. Out of the environmental and anthropogenic factors assessed (excluding these background variables), seasonal temperatures (i.e., winter temperature and maximum spring temperature) were the most important predictors of establishment rates (Figure 5; Appendix S3: Table S1). Winter temperature was the most important predictor of waiting time in the range‐wide best fit model. Our results indicate that quadrats with lower winter temperatures had longer waiting times, an effect that is strongest in the Northern mixed wood shield, with less importance in the Central plains ecoregion, and no importance in the Southeastern forests and plains ecoregion. Maximum temperature in the spring was the second most important factor in the full model, with a lengthening effect on waiting times in warmer localities. Maximum spring temperature displayed subregional differences in its effect on waiting times, with a shortening effect on waiting time in the northern subregion, but lengthening effects in the central and southeastern subregions. Anthropogenic fragmentation had a lengthening effect on waiting times in the full range model, as well as in the northern model and the southeastern model.

Other factors not included in the best fit full range model were found to be important in particular subregions. Average summer precipitation had a positive effect on waiting time for quadrats in both the northern and central models. Elevation was included in the northern and southeastern models, albeit demonstrating opposite effects in these two subregions, a shortening effect in the north and a lengthening effect in the southeast. Human population density was found to have a relatively small shortening effect on waiting time only in the central subregion.

The four models showed a strong ability to explain waiting times. Based on the measures of goodness of fit, marginal and conditional *R* ^2^, all four models explained over 50% of the variance in waiting times (Table 2). However, when focusing on the variance explained by the environmental and anthropogenic variables alone (i.e., excluding the background variables, average waiting time of neighborhood and year of first detection), the four models performed quite differently. In the range‐wide, central, and southeastern models, the drivers of range expansion explained only 7%, 7%, and 5% of the variance in waiting times, respectively. In the northern mixed wood shield, however, these factors were found to have a stronger effect as they explained a much higher percentage of the variance (22%).

**TABLE 2:** Measures of proportion of variance explained by the four mixed‐effects linear regression models

	*R* ^2^_marginal_	*R* ^2^_conditional_	*R* ^2^_marginal_ of drivers
Range‐wide	0.63	0.75	0.07
Northern mixed wood shield	0.66	0.82	0.22
Central plains	0.71	0.75	0.07
Southeastern forests and plains	0.58	0.65	0.05

## DISCUSSION

We sought to gain a better understanding of the roles of various environmental and anthropogenic habitat characteristics on *L. dispar* establishment rates across the US invasive range using a macroscale approach, with the purpose of contributing to the prioritization of treatment of problem areas and of furthering our knowledge on the role of local invasion drivers on regional patterns in spatial spread. Our examination of waiting times to establishment across the invasive range showed that there is no appreciable geographic difference in the average number of years to establishment. This pattern is particularly interesting, as it suggests that localized spread differs spatially from patterns of range‐wide expansion. Previous research demonstrates that the rate of range expansion is greater in the northern portion of the range compared to the southern portion (Tobin et al., 2007). The contrasts in these observed patterns highlight the need for multi‐scale investigations (i.e., different spatial grain and extents) in order to gain a more complete understanding of the patterns and processes of biological invasions.

A visual assessment of spatial trends of waiting times appears to indicate an increase in waiting times across the leading edge, suggesting that local rates of establishment of *L. dispar* may be currently decelerating. Furthermore, spatial patterns of decelerating establishment rates coincide with the boundaries of the STS program from 2007 to 2015, potentially evidencing the success of *L. dispar* management programs to slow regional spread rates even with the exclusion of treatment blocks from our data set. A formal assessment of the effects and efficacy of STS treatments on large‐scale patterns of *L. dispar* spread would be a worthwhile pursuit for future research efforts.

**FIGURE 6:**
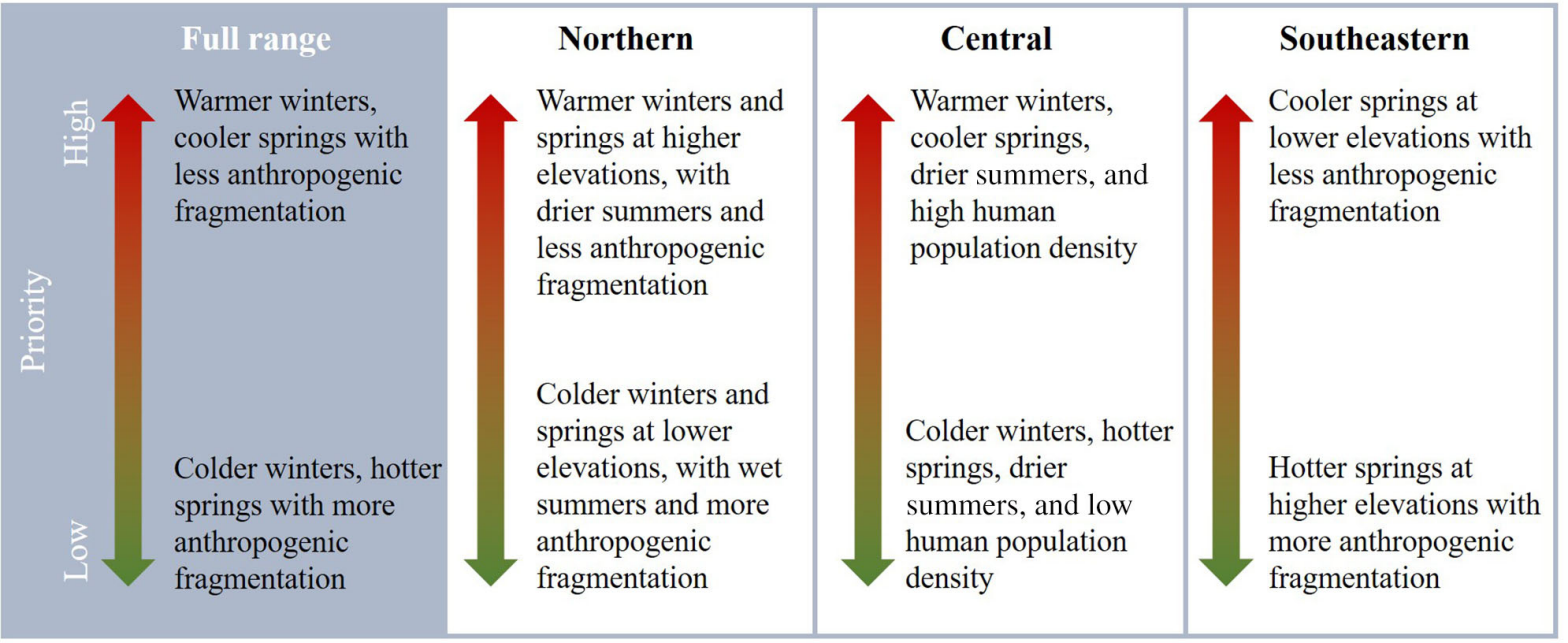
Recommended prioritization guidelines informed by our findings regarding the effects of local drivers of *L. dispar* landscape spread. Each panel delineates factors that increase or decrease establishment rates as additive effects in order to characterize high or low priority conditions for treatment for the full transition zone and each subregion

Our study allowed us to identify the different environmental and anthropogenic habitat characteristics that accelerate *L. dispar* establishment rates in each subregion within the transition zone, allowing us to characterize areas that may be particularly vulnerable and therefore should be considered as high priority for treatment (Figure 6). These insights could be used to define region‐specific criteria for the prioritization of vulnerable areas for treatment. Potential problem areas are currently identified and prioritized by the STS program using a decision algorithm that assigns priority indices to potential problem areas across the transition zone by taking into account four primary components: the population density of *L. dispar* in the identified area in the current and previous year, the distance of the problem area from infested areas, the “background population density” of *L. dispar* in the surrounding area, and the location relative to other problem areas being evaluated (Tobin & Sharov, 2007). Including information pertaining to environmental and anthropogenic drivers, in addition to the population density components currently included, in the prioritization process or the decision algorithm has the potential to improve the identification and prioritization of potential problem areas, and consequently improve efforts to slow invasive spread of *L. dispar*.

When taking into consideration the effects of the background variables, seasonal temperatures were the top predictors for establishment rates. Low winter temperatures were the best predictor for local invasion cold spots in the full range model; a pattern that was also observed in the northern and central models, but not in the southeast. Low winter temperatures influence *L. dispar* population growth and survival through multiple mechanisms. First, if winter temperatures get too cold, as occurs in northern and central subregions, eggs may face increased overwintering mortality due to freezing (Bess, 1961; Madrid & Stewart, 1981; Streifel et al., 2019). Furthermore, suboptimal temperatures may delay egg hatch and slow larval development, which may result in late oviposition and low reproductive success (Gray, 2004; Walter, Meixler, et al., 2015b).

Maximum spring temperatures were the second most important environmental factor in creating local hot/cold spots of invasion in all subregions, as well as the full range model. The strongest positive effect was in the southeast subregion (warmer temperatures resulted in longer waiting times to establishment), which includes the southernmost edge of the invaded range, although a weaker positive effect was also detected in the central subregion. Conversely, in the northern subregion where temperatures tend to be colder in general, a negative relationship between maximum spring temperatures and waiting times to establishment suggest that warmer springtime temperatures in this region create a more favorable environment for *L. dispar*, likely to accelerate development and improve reproductive success. Exposure to supra‐optimal temperatures during early larval stages, particularly in the warmer Southeast, may impact *L. dispar* physiological processes, which may in turn decrease population growth rates (Faske et al., 2019; May et al., 2018; Thompson et al., 2017; Tobin et al., 2014). Our findings support the notion that observed patterns of range retraction in the Southeast may be caused by supra‐optimal temperatures (Tobin et al., 2014) and may be the first evidence that supra‐optimal temperatures suppress spread in the Central region.

In addition to seasonal temperatures, anthropogenic fragmentation appeared to make quadrats less vulnerable to *L. dispar* invasion at the full range level, and in the Northern and Southeastern subregions. These patterns are expected, as poor matrix quality and decreased landscape connectivity as a result of human land use increases dispersal mortality of ballooning larvae and pedestrian egg‐bearing *L. dispar* adults (Vandermeer et al., 2001; Walter et al., 2016). Elevation, summer precipitation, and human population density were also significant drivers of local spread, albeit only at the subregional level. Elevation was a contributing predictor in the northern and southeastern models; however, with contradicting effects. Increased elevation made quadrats less vulnerable in the southeast, but more vulnerable in the northern subregion. Our results suggest that discrepancies may also be caused by the confounding influence of differing geographical contexts. For instance, the divergent effects of elevation may be due to the fact that topography is more homogenous in the northern region than in the southeastern Appalachian forests, where *L. dispar* populations in high elevation areas experience higher risk of extreme winter low temperatures than the rest of the southeast (J. A. Walter, unpublished data). Summer precipitation made quadrats less vulnerable, albeit only in the northern and central plains models. High precipitation in the summer is detrimental to *L. dispar* population growth, as rainy conditions may hinder the mobility of larvae or even wash them away (D'Amico & Elkinton, 1995; Leonard, 1974; Pernek et al., 2008). Furthermore, the fungus *Entomophaga maimaiga*, which infects neonates of *L. dispar*, performs best in cool moist environments (Reilly et al., 2014; Weseloh et al., 1993), suggesting a possible interaction in the effects of temperature and precipitation on waiting time to establishment of this widespread pathogen, a potential interaction that could be further investigated in future studies. Human population density was a significant predictor of waiting times in the central plains subregion exclusively, displaying a small shortening effect on waiting times. In the central plains, higher levels of human population density may represent increased anthropogenically driven propagule pressure where high fragmentation restricts natural dispersal (Bigsby et al., 2011).

The strength of the ability of these environmental and anthropogenic drivers to predict local *L. dispar* establishment rates exhibited spatial trends, suggesting that the value of our prioritization guidelines derived from our findings may vary among subregions. While these drivers were found to be relatively weak predictors of waiting times in the full range model, as well as the central plains and southeastern models, the variance explained by these drivers in the northern subregion was much higher. This pattern indicates that the implementation of the STS decision algorithm for prioritization of problem areas could have the most benefit in the northern subregion by accounting for the influence of the drivers identified for this region (winter temperatures and elevation in particular). Furthermore, this finding suggests that the processes underlying *L. dispar* establishment rates in the northern part of the US range are different from the rest of the transition zone, emphasizing the need to take into consideration subregional differences when prescribing management. Observed differences in the north coincide with observed geographic differences in the frequency of *L. dispar* defoliating outbreaks, a known driver of pulsed invasive spread (Johnson et al., 2006; Walter, Johnson, et al., 2015a). Per outbreak reports compiled by the USDA Forest Service, the area of forest defoliated by *L. dispar* is much lower in northern states than in the rest of the invasive range, emphasizing the role of other drivers of local establishment rates besides pulses in population growth (USDA Forest Service, 2020a).

The findings of this study demonstrate the utility of macroscale investigations in producing findings that are relevant to large‐scale management of invasive species. Performing our study at this spatial grain and extent has allowed us to both differentiate the role of various factors in local *L. dispar* establishment and describe spatially dependent relationships across the transition zone. We were able to identify local characteristics that increase local establishment rates of *L. dispar* at both the subregional and range‐wide levels. Future research efforts should build upon our spatially expansive insights by taking advantage of the long temporal span of the STS data set, and in turn focus on understanding the temporal dynamics of forest insect invasions. For example, the role of temporal variability of drivers, such as temperature and precipitation, on establishment rates is little understood. Particularly, future studies should investigate whether finer‐scaled temporal factors, such as year‐to‐year variability or above/under average values in a given year, have a significant effect on waiting times to establishment.

Furthermore, being able to discern subregional patterns has allowed us to identify potential implications of climate change on the management of invasive species. We found that winter temperatures are the strongest limits to localized spread, particularly in the northern parts of the range. Our results demonstrate that variation in mean winter temperatures across 5 × 5 km quadrats within a single region can explain variation in establishment rates, even when accounting for propagule pressure from neighboring quadrats. Increasing global temperatures may lessen the strength of these limits, potentially resulting in faster establishment rates in the northern leading edges, as well as changes in the hierarchy of these drivers. Similarly, fluctuating precipitation levels due to a changing climate has the potential to influence the vulnerability of areas to *L. dispar*, as well as alter the relative importance of these drivers. An understanding of the interacting effects of local climatic factors on changes in species distributions has never been a more urgent research priority. Similar macroscale studies could be performed to improve understanding of the role of local climate on the invasion dynamics of other invasive forest insects.

## CONFLICT OF INTEREST

The authors declare no conflict of interest.

## Supporting information


Appendix S1

Appendix S2

Appendix S3

## Data Availability

Catch data for *Lymantria dispar* used to generate waiting time to establishment data in this study were provided by the USDA‐sponsored Slow‐the‐Spread Program; these data are available on their website and are available in the Knowledge Network for Biocomplexity at https://doi.org/10.5063/9G5K74. The data on environmental and anthropogenic characteristics across the range were obtained from sources cited in Table 1; these data are available in the Knowledge Network for Biocomplexity at https://doi.org/10.5063/Z60MGX.
