# Supplementary material for: Assessing drivers of localized invasive spread to inform large‐scale management of a highly damaging insect pest
Source: Ecol Appl. 2022 Feb 20;32(3):e2538. doi: 10.1002/eap.2538 (PMC9286796; doi:10.1002/eap.2538)
Supplement: Supplementary file 1 — Appendix S1 [file EAP-32-0-s002.pdf]

Supporting Information. Nunez-Mir, G. C., Walter, J. A., Grayson, K. L., and Johnson, D. M. Assessing drivers of localized invasive spread to inform large-scale management of a highly damaging insect pest. Ecological Applications.

Appendix S1

Table S1: Pearson's *r* for correlations between observed and predicted values of the number of *L. dispar* present in each 5 by 5 km quadrat on a given year. Correlations were performed for each year separately to validate Bayesian structural time series model performance.

Year	*Correlation coefficient (Pearson's* r*)*
1995	0.45
1996	0.16
1997	0.75
1998	0.90
1999	0.92
2000	0.86
2001	0.77
2002	0.83
2003	0.61
2004	0.69
2005	0.50
2006	0.58
2007	0.83
2008	0.55
2009	0.38
2010	0.68
2011	0.45
2012	0.62
2013	0.76
2014	0.73
2015	0.54

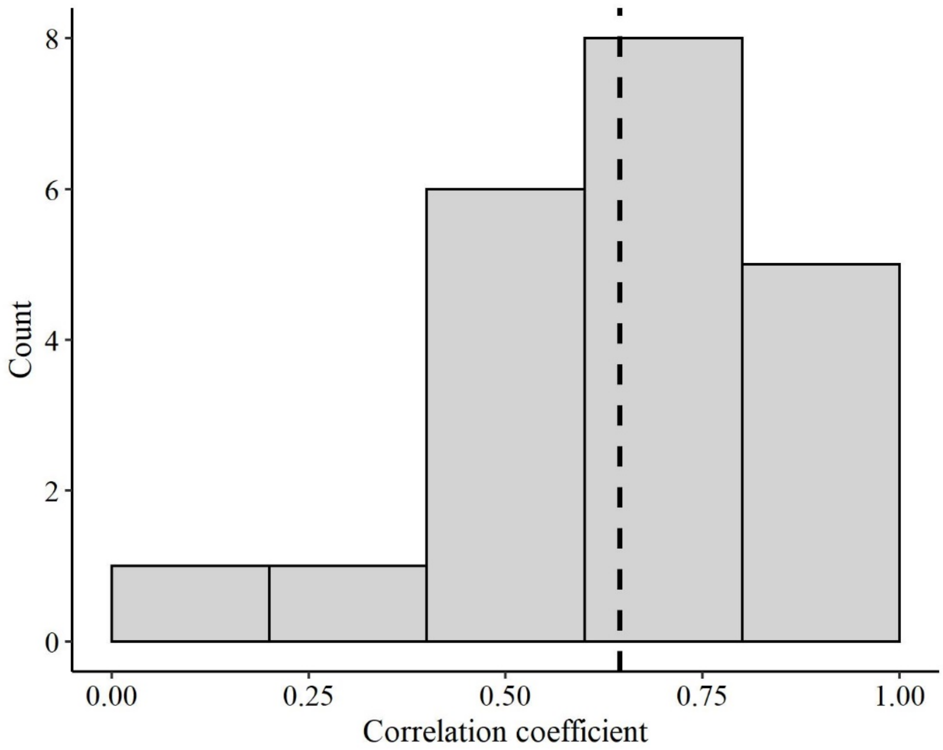

Figure S1: Histogram summarizing the coefficients (Pearson's *r*) of correlations between observed and predicted values for each year analyzed (1995-2015). The average correlation coefficient, depicted by a dashed line, was 0.65.
