# Supplementary material for: Assessing drivers of localized invasive spread to inform large‐scale management of a highly damaging insect pest
Source: Ecol Appl. 2022 Feb 20;32(3):e2538. doi: 10.1002/eap.2538 (PMC9286796; doi:10.1002/eap.2538)
Supplement: Supplementary file 2 — Appendix S2 [file EAP-32-0-s003.pdf]

Supporting Information. Nunez-Mir, G. C., Walter, J. A., Grayson, K. L., and Johnson, D. M. Assessing drivers of localized invasive spread to inform large-scale management of a highly damaging insect pest. Ecological Applications.

Appendix S2

Table S1: Variance inflation factors for the four mixed-effects regression models presented in Figure 5.

Model	*Variable*	*VIF*
Full model	Mean winter temperature	1.70
	Maximum spring temperature	1.74
	Anthropogenic fragmentation (st. dev.)	1.01
	Average waiting time of neighborhood	1.04
	Year of initial detection	1.02
Northern mixed	Mean winter temperature	2.84
	Maximum spring temperature	1.05
	Anthropogenic fragmentation (mean)	1.09
	Elevation	2.40
	Summer precipitation	1.63
	Average waiting time of neighborhood	1.25
	Year of initial detection	1.32
Central plains	Mean winter temperature	1.24
	Maximum spring temperature	1.36
	Summer precipitation	1.30
	Human population density	1.07
	Average waiting time of neighborhood	1.13
	Year of initial detection	1.15
Southeastern forests and plains	Maximum spring temperature	4.48
	Anthropogenic fragmentation (st. dev.)	1.05
	Elevation	4.24
	Average waiting time of neighborhood	1.10
	Year of initial detection	1.15
