# Supplementary material for: Assessing drivers of localized invasive spread to inform large‐scale management of a highly damaging insect pest
Source: Ecol Appl. 2022 Feb 20;32(3):e2538. doi: 10.1002/eap.2538 (PMC9286796; doi:10.1002/eap.2538)
Supplement: Supplementary file 3 — Appendix S3 [file EAP-32-0-s001.pdf]

Supporting Information. Nunez-Mir, G. C., Walter, J. A., Grayson, K. L., and Johnson, D. M. Assessing drivers of localized invasive spread to inform large-scale management of a highly damaging insect pest. Ecological Applications.

Appendix S3

Table S1: Detailed results of the four mixed-effects regression models presented in Figure 5.

Model	*Variable*	*Estimate*	*SE*
Full Range	Mean winter temperature	-0.0611	0.0087
	Maximum spring temperature	0.0219	0.0054
	Anthropogenic fragmentation (st. dev.)	0.0115	0.0025
	Average waiting time of neighborhood	0.2008	0.0023
	Year of initial detection	-0.1258	0.0032
Northern mixed	Mean winter temperature	-0.1417	0.0135
	Maximum spring temperature	-0.0326	0.0048
	Anthropogenic fragmentation (mean)	0.0173	0.0032
	Elevation	-0.0737	0.0093
	Summer precipitation	0.0472	0.0056
	Average waiting time of neighborhood	0.1751	0.0045
	Year of initial detection	-0.2624	0.0071
Central plains	Mean winter temperature	-0.0439	0.0072
	Maximum spring temperature	0.0239	0.0045
	Summer precipitation	0.0239	0.0042
	Human population density	-0.0094	0.0026
	Average waiting time of neighborhood	0.1815	0.0035
	Year of initial detection	-0.1084	0.0046
Southeastern forests and plains	Maximum spring temperature	0.0596	0.0113
	Anthropogenic fragmentation (st. dev.)	0.0204	0.0042
	Elevation	0.069	0.0118
	Average waiting time of neighborhood	0.2008	0.0044
	Year of initial detection	-0.084	0.0049
